# Supplementary material for: Influenza A Virus Infection Reactivates Human Endogenous Retroviruses Associated with Modulation of Antiviral Immunity
Source: Viruses. 2022 Jul 21;14(7):1591. doi: 10.3390/v14071591 (PMC9320126; doi:10.3390/v14071591)
Supplement: Supplementary file 1 [file viruses-14-01591-s001.zip › MotifAnalysis.zip!/MotifOutput_SC35MdNS1/homerResults/motif6.similar.html]

motif6

## Information for motif6

C
G
T
A
A
G
T
C
A
C
G
T
A
G
T
C
A
C
G
T
A
C
T
G
A
C
G
T
C
G
T
A
A
C
G
T
A
G
T
C
  
Reverse Opposite:  

A
C
T
G
G
T
C
A
C
G
A
T
G
T
C
A
A
G
T
C
C
G
T
A
A
C
T
G
C
G
T
A
A
C
T
G
A
C
G
T
  

|  |  |
| --- | --- |
| p-value: | 1e-55 |
| log p-value: | -1.271e+02 |
| Information Content per bp: | 1.944 |
| Number of Target Sequences with motif | 34.0 |
| Percentage of Target Sequences with motif | 44.16% |
| Number of Background Sequences with motif | 245.3 |
| Percentage of Background Sequences with motif | 0.54% |
| Average Position of motif in Targets | 144.2 +/- 43.9bp |
| Average Position of motif in Background | 97.4 +/- 82.2bp |
| Strand Bias (log2 ratio + to - strand density) | 10.0 |
| Multiplicity (# of sites on avg that occur together) | 1.09 |
| Motif File: | file (matrix) reverse opposite |

### Similar de novo motifs found

|  |  |  |  |  |  |  |  |
| --- | --- | --- | --- | --- | --- | --- | --- |
| Rank | Match Score | Redundant Motif | P-value | log P-value | % of Targets | % of Background | Motif file |
| 1 | 0.897 | C G T A A G T C A C G T A G T C A C G T A C T G A C G T C G T A | 1e-47 | -108.665102 | 45.45% | 1.07% | motif file (matrix) |
| 2 | 0.694 | A C T G A G T C C G T A A G T C A C G T A G T C A C G T A C T G | 1e-35 | -81.513951 | 37.66% | 1.15% | motif file (matrix) |
| 3 | 0.625 | C G T A A T G C A C G T A G T C A C G T A C T G A C T G A C G T | 1e-6 | -16.071801 | 10.39% | 0.73% | motif file (matrix) |
